# Application of PCR-based approaches for evaluation of cell-free DNA fragmentation in colorectal cancer

**DOI:** 10.3389/fmolb.2023.1101179

**Published:** 2023-03-27

**Authors:** Anastasia P. Koval, Alexandra S. Khromova, Konstantin A. Blagodatskikh, Yulia V. Zhitnyuk, Yanina A. Shtykova, Aleksandr A. Alferov, Nikolay E. Kushlinskii, Dmitry S. Shcherbo

**Affiliations:** ^1^ Institute of Translational Medicine, Pirogov Russian National Research Medical University, Moscow, Russia; ^2^ Center of Genetics and Reproductive Medicine “Genetico”, Moscow, Russia; ^3^ Federal Center for Brain and Neurotechnology, Moscow, Russia; ^4^ Laboratory of Clinical Biochemistry, N. N. Blokhin Cancer Research Medical Center of Oncology, Moscow, Russia

**Keywords:** cell-free DNA, liquid biopsy, PCR, digital droplet PCR, colorectal cancer

## Abstract

Cell-free DNA (cfDNA) testing is the core of most liquid biopsy assays. In particular, cfDNA fragmentation features could facilitate non-invasive cancer detection due to their interconnection with tumor-specific epigenetic alterations. However, the final cfDNA fragmentation profile in a purified sample is the result of a complex interplay between informative biological and artificial technical factors. In this work, we use ddPCR to study cfDNA lengths in colorectal cancer patients and observe shorter and more variable cfDNA fragments in accessible chromatin loci compared to the densely packed pericentromeric region. We also report a convenient qPCR system suitable for screening cfDNA samples for artificial high molecular weight DNA contamination.

## 1 Introduction

Circulating cell-free DNA (cfDNA) can reveal insights on physiological and pathological conditions, including pregnancy (Barrett et al., 2011), cancer (Heitzer et al., 2015), inflammation (van der Meer et al., 2019) and transplant rejection (Bloom et al., 2017; Thongprayoon et al., 2020), among others. At the same time, cfDNA analysis is technically challenging due to low concentrations and susceptibility to inconsistencies in the preanalytical stages, including artificial contamination with high molecular weight (HMW) DNA from damaged blood cells that can mask native cfDNA features (Bartak et al., 2019; Meddeb et al., 2019; van der Pol et al., 2022). In particular, the lengths and endpoint distributions of cell-free DNA fragments appear to relate to chromatin structure in the cells of origin, making a connection from cfDNA fragmentation features to a functional state of the genome. This phenomenon is extensively studied with high-throughput sequencing that provides a versatile methodological framework (Ivanov et al., 2015; Snyder et al., 2016; Cristiano et al., 2019; Lo et al., 2021). At the same time, PCR-based approaches remain widespread and more cost-effective in some cfDNA analysis applications. Most of them focus on sample quality control and target various protein-coding genes (Devonshire et al., 2014; Fernando et al., 2018; Alcaide et al., 2020) or multicopy repetitive elements (Rago et al., 2007; Saelee et al., 2022). Beyond that, there are studies suggesting the DNA integrity index that is determined with qPCR as a diagnostic and prognostic biomarker of colorectal cancer (Umetani et al., 2006; Hao et al., 2014; El-Gayar et al., 2016). Here, we explore the utility of PCR for cfDNA fragment size analysis in two modalities: 1) The evaluation of cfDNA fragment sizes in cancer-specific open-chromatin regions (OCRs) from patients with colorectal cancer (CRC) using droplet digital PCR and 2) the detection of artificial high molecular weight genomic DNA contamination in cfDNA samples using quantitative PCR.

## 2 Methods

### 2.1 Patient characteristics

The study was endorsed by the Local Ethics Committee of the Pirogov Russian National Research Medical University. Each participant signed written informed consent. Blood samples were prospectively collected from healthy donors and previously untreated individuals with CRC. A cohort of 85 participants included two groups with and without CRC, balanced by age and sex. CRC examination and treatment were carried out at the N.N. Blokhin Medical Research Center of Oncology. Samples from the apparently healthy subjects were collected at the Federal Center for Brain and Neurotechnology. Clinical and radiological diagnosis of CRC patients was confirmed by morphological examination of the tumor according to WHO classification, which revealed adenocarcinoma of varying degrees of differentiation.

### 2.2 Blood and cfDNA processing

For extraction of cell-free DNA, 9 mL of blood was collected in GBM scf-DNA tubes (GRADBIOMED, Moscow, Russia). Plasma was separated immediately after collection by double centrifugation (1,900 g, room temperature for 15 min, then 16,000 g +4°C for 10 min), then cfDNA samples were extracted from the total plasma volume obtained according to the manufacturer’s protocol using the QIAamp MinElute ccfDNA Midi Kit (QIAGEN). Cell-free DNA was eluted in 30 μl of ultrapure water. The cfDNA samples obtained were quantified using the Qubit dsDNA HS Assay Kit (Thermo Fisher Scientific) and stored at −20°C prior to PCR analysis. Automated electrophoresis for length detection and control of sample DNA was performed using 2200 TapeStation System, Agilent, with High Sensitivity D1000 tapes and reagents.

### 2.3 Digital droplet PCR

The ddPCR system targets three genomic regions (OCR1, OCR2, and CCR). We designed one universal primer and two opposing primers (to generate amplicons of different lengths) and one TaqMan probe for each region. The probes contained FAM or HEX fluorophores for multiplexing. The sequences of oligonucleotides are provided in Supplementary Table S1. Primers and probes were manufactured by Evrogen (Moscow, Russia). Each PCR contained 10 pmol of primers and a probe, 1X ddPCR Supermix for probes (no dUTP) (Bio-Rad), water, and sample DNA. The equivalent portion (9.4 of 30 μl for both long and short targets) of each cfDNA sample was used for analysis. The reactions were performed using the Bio-Rad QX200 ddPCR system.

### 2.4 Standard samples

For calibration of the contamination assay, fragmented and unfragmented DNA samples obtained from the Raji cell line were used as standards. Fragmented samples were prepared using the Covaris ultrasonic fragmentation system (150 bp mode for 15 min). The average length of the fragments after ultrasound shearing was approximately 170 bp. Then, both fragmented and unfragmented DNA samples were diluted to 1 ng/μl and mixed to obtain samples with 1%, 5%, 25%, and 50% unfragmented DNA mass. The final DNA concentration in the standard samples was confirmed using the Qubit fluorometer.

### 2.5 Real-time PCR

We designed real-time PCR system to target multi-copy genomic regions (long non-coding RNA genes) presented on several chromosomes. Two primer pairs generate 106 bp and 612 bp amplicons that do not overlap. The amplification process could be monitored using TaqMan probes with FAM fluorophore for the 106 bp amplicon and HEX for the 612 bp amplicon. The sequences of oligonucleotides are provided in Supplementary Table S1. The system can potentially be used in multiplex format, but to avoid imbalance in the amplification efficiency of short and long amplicons, we used it in monoplex format. Primers and probes were manufactured by Evrogen (Moscow, Russia). Each PCR contained 5 pmol of primers and a probe, 1X HS Taq DNA mix (Evrogen, Moscow, Russia), water and sample DNA (1 ng). Amplification was performed in BioRad CFX96 with the following program: 95°C 3 min, (95°C 30 s, 56°C 30 s, and 72°C 30 s) 40 cycles. The contamination score was calculated as the ratio of short and long amplicons. We performed ∆∆Ct analysis in BioRad CFX Manager software to calculate the ratios, selecting a 106 bp amplicon as a reference gene. The percentage of contamination was calculated as Relative Normalized Expression * 50% (control sample). Sample qPCR data are available in Supplementary Material with annotation in the Supplementary Text.

## 3 Results

### 3.1 Fragment lengths of cfDNA in tumor-specific open-chromatin regions

**FIGURE 1:**
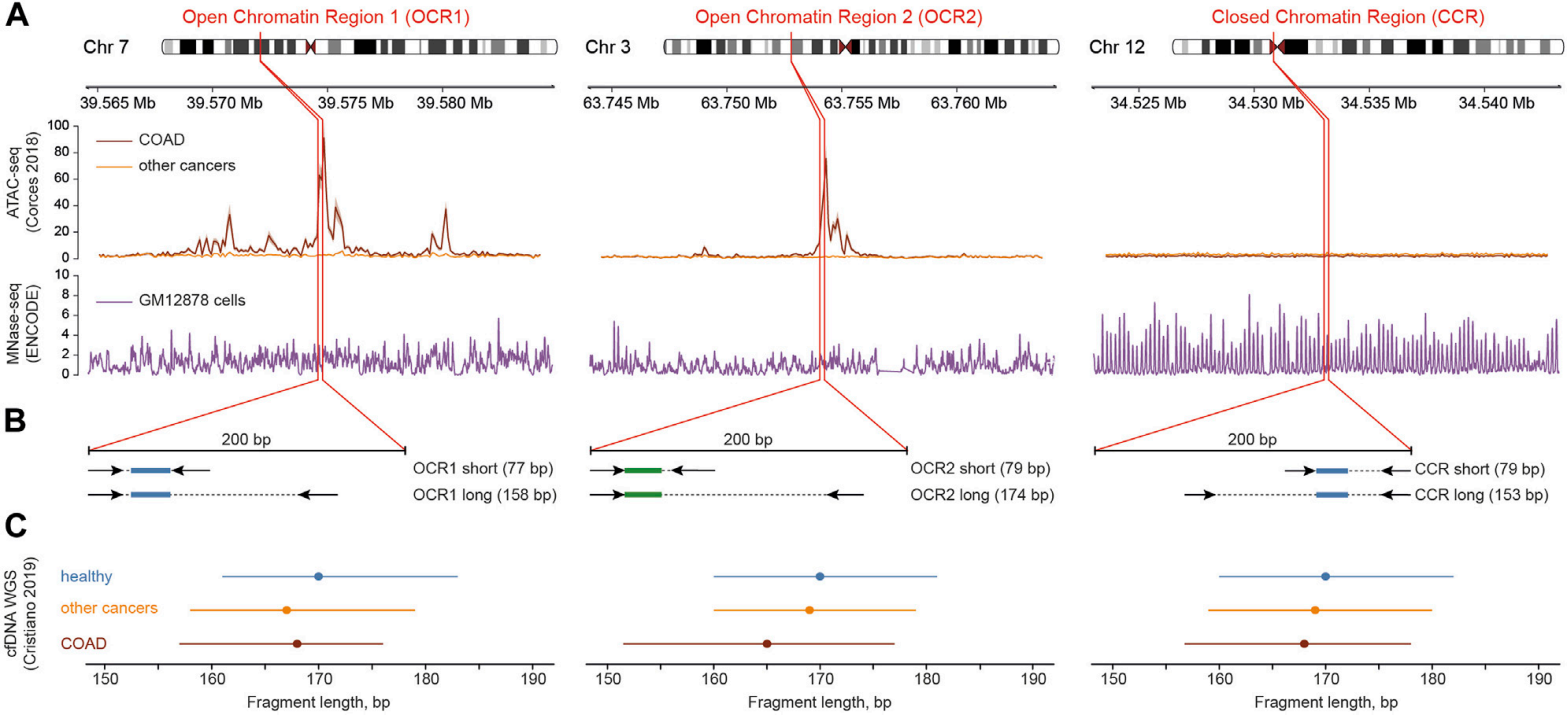
Outline of the design of ddPCR systems. **(A)** Chromatin state (ATAC-seq, top track) and nucleosome positioning (MNase-seq, bottom track) context near the studied genomic regions (red borders) in cancer and normal cells. Data from (Bernstein et al., 2012; Corces et al., 2018). **(B)** Positioning of primers (arrows) and probes (colored rectangles: green for labeled with HEX and blue for labeled with FAM fluorescent dyes) within the target regions. **(C)** Length of cfDNA fragments shown as medians (dots) and interquartile ranges (lines) derived from whole-genome sequencing data of healthy and cancer individuals (Cristiano et al., 2019).

**FIGURE 2:**
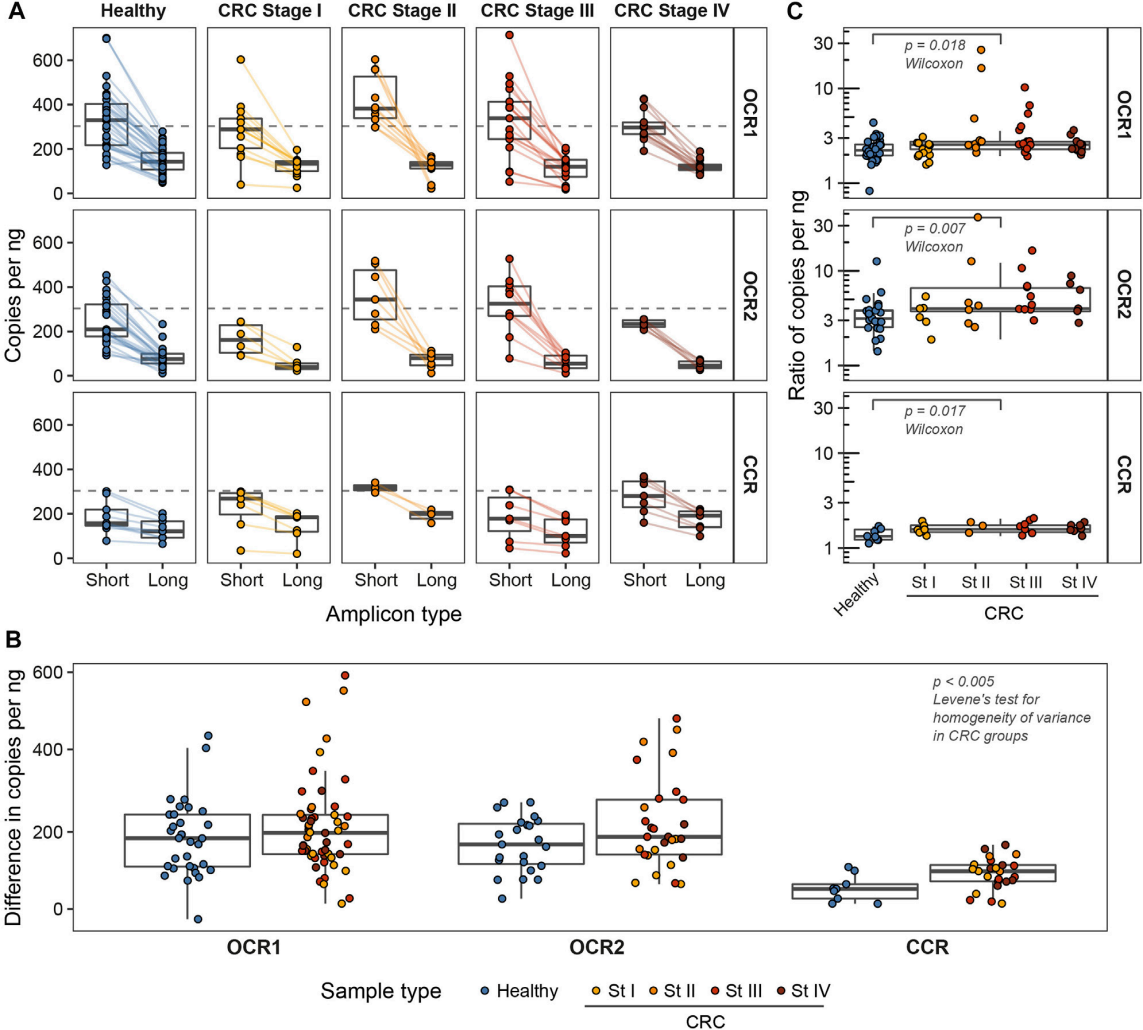
The number of cfDNA copies of studied lengths per nanogram of input DNA in OCR1, OCR2, and CCR. **(A)** The number of cfDNA copies estimated from ddPCR. Colored lines connect the two estimates for a single sample. Dashed lines mark the theoretical number of copies in 1 ng of human genomic DNA (approx. 303 copies/ng). **(B)** Ratios of numbers of short to long cfDNA copies per nanogram. **(C)** Differences in absolute numbers of short and long cfDNA copies per nanogram.

We focused on the two genomic loci where chromatin in colorectal adenocarcinoma (COAD) cells predominantly exists in open conformation (open chromatin regions, OCR1 and OCR2) (Corces et al., 2018). This may lead to aberrant cfDNA fragmentation in colorectal cancer patients according to our previous report (Zhitnyuk et al., 2022). For comparison, we selected a locus with stable nucleosome positioning in the pericentromeric region of the 12th chromosome (closed chromatin region, CCR) (Figure 1A). We extracted cfDNA fragment sizes from the whole-genome sequencing dataset (Cristiano et al., 2019) and found that the median cfDNA lengths of from healthy individuals in all three regions were 170 bp while medians of cfDNA lengths in colorectal cancer patients varied, with the largest difference between CRC and healthy individuals in OCR2 (Figure 1C). We designed ddPCR systems to compare the lengths of cfDNA molecules in the selected loci (Figure 1B) in cfDNA samples from healthy donors (*n* = 32) and CRC patients (*n* = 53). Reportedly, the major fraction of cfDNA is ∼160–170 bp in length and is associated with mononucleosomal DNA wrapping (Snyder et al., 2016; Sanchez et al., 2021). To detect changes in DNA fragmentation caused by differences in nucleosome positioning the ddPCR systems design implied amplification of two types of products: “short” <80 bp and “long” >150 bp. For each sample, both types of amplicons were amplified in two of the three target regions, due to the limited availability of cfDNA. Digital droplet PCR enables absolute quantification of DNA copy numbers, i.e., fragments that are equal to or longer than the respective target lengths in this case. Amplification of short products allows quantification of nearly total number of cfDNA copies while longer ones correspond to mononucleosomal length. Nucleosomes are depleted in open chromatin regions and provide less protection for DNA what might lead to a higher proportion of cfDNA fragments shorter than 150 bp. Decreased DNA integrity would result in a steeper decline in copy numbers from short to long products, while less fragmented DNA would result in a less pronounced difference (Figure 2A). We found that the medians of differences in copy numbers between short and long targets for healthy and cancer samples were 52.4 (SD = 32.9) and 98.2 (SD = 40.5) in the CCR, 184 (SD = 97.4) and 198 (SD = 122) in the OCR1, 168 (SD = 70.1) and 188 (SD = 118) in the OCR2, respectively (Figure 2B). The variances of the differences in the CRC samples were lower in the CCR (SD_CCR_ = 41) compared to both OCRs (SD_OCR1_ = 123, SD_OCR2_ = 119; *p* < 0.005, Levene’s test for homogeneity of variance across groups). This may indicate that the cfDNA sizes are less stable across samples in OCRs compared to the pericentromeric CCR. Furthermore, we found that the ratio of short to long copy numbers per nanogram of DNA was higher in all three regions of CRC, indicating an increased abundance of shorter cfDNA fragments in cancer patients (*p* < 0.05, Wilcoxon test) (Figure 2C).

### 3.2 Cell-free DNA contamination screening

**FIGURE 3:**
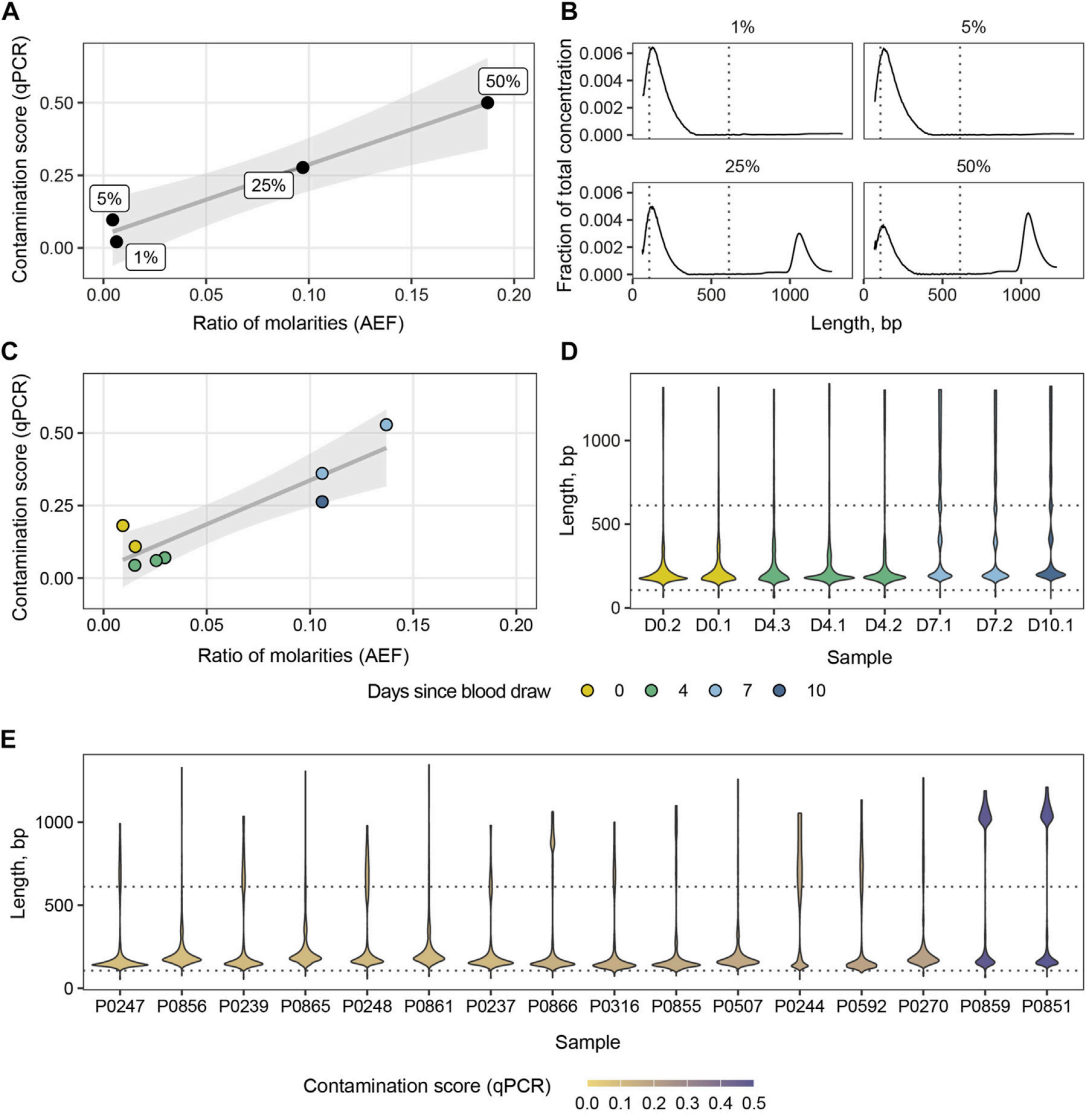
Ratios of molarities derived from AEF data versus contamination scores computed from qPCR for the panel of standard samples **(A)** and cfDNA samples stored for various periods of time in collection tubes **(C)**. Distribution of cfDNA concentration for the panel of standard samples **(B)**. Violin plots of cfDNA concentration distributions for blood samples stored for various periods of time **(D)** and a set of clinical samples **(E)**. Dotted lines indicate amplicon lengths.

Another potential application of the ability to detect variations in cfDNA length with PCR is to screen samples for the presence of HMW DNA. These longer fragments likely originate from blood cells destroyed during blood collection or during other preanalytical stages. They mask tumor-specific alterations in ctDNA eventually interfering with the downstream analysis (Saelee et al., 2022). To be applicable in the clinical setting, the screening system should be inexpensive, rapid and require small amounts of cfDNA. We followed the established strategy and designed a qPCR system targeting multicopy non-coding RNA genes to increase the copy number of the target. BLAST search revealed about 25 annealing sites of the primer pair in human genome (hg19) with amplicons of two lengths: 106 bp and 612 bp. This allowed us to perform qPCR reactions starting at 1 ng of input DNA. First, we generated a panel of standard samples with known mass fractions of HMW DNA: 50%, 25%, 5%, and 1%. We used the panel to calculate contamination score and compare it to automated electrophoresis (AEF) data (Figures 3A, B). The contamination score was calculated using the ∆∆Ct method based on qPCR results, while the ratios of molarities of the fragments longer than 106 bp and 612 bp were based on AEF-derived DNA distributions. Next, we placed eight blood samples from two healthy donors into the preservative-containing collection tubes and stored them for 2, 4, 7, and 10 days before the cfDNA purification and further analysis. The collection tubes are designed to protect cfDNA from HMW contamination for 7 days. After this period, we observed an expected increase in both qPCR contamination scores and AEF molarity ratios, and qPCR estimates were in agreement with AEF data (Pearson *R* ^2^ = 0.91, 95% CI 0.56–0.98, *p* < 0.005) (Figure 3C). Notably, after 1 week of blood storage the cfDNA distribution shows peaks at 360–400 bp and 540–600 bp corresponding to DNA lengths wrapping two and three nucleosomes (Figure 3D). This could be indicative of the destruction of nucleated blood cells in the course of apoptosis during sample storage. Finally, we examined a set of cfDNA samples routinely processed in our laboratory for HMW DNA contamination and found elevated HMW DNA levels in two of 16 samples, which was confirmed by AEF (Figure 3E).

## 4 Discussion

Numerous PCR-based techniques provide relatively inexpensive, widely adopted and yet powerful tools for nucleic acid research. Relative comparison of the DNA fragment lengths is one of the potential applications. Circulating cell-free DNA in blood plasma is an attractive source of genomic and epigenomic biomarkers with fragmentation features being one of the dimensions. Various biological factors have been shown to alter cfDNA fragmentation, including the positioning of nucleosomes and chromatin state in cells of origin (Ivanov et al., 2015; Snyder et al., 2016; Bronkhorst et al., 2022; van der Pol et al., 2022). Previous studies revealed higher fragmentation of tumor-derived cfDNA fragments and their enrichment with somatic mutations, particularly in colorectal cancer patients (Diehl et al., 2005; Mouliere et al., 2011; 2013), while at the same time some evidence conversely support the presence of longer molecules in tumor cfDNA fraction (Umetani et al., 2006; Ganesamoorthy et al., 2022). The complexity of cfDNA size profile seems to be the result of a balance between several biological processes including apoptosis, necrosis, senescence and active release that may be altered in pathology (Rostami et al., 2020; Ungerer et al., 2021; Ungerer et al., 2022). Nevertheless, the exact processes that determine changes in cfDNA cleavage and lead to aberrant fragmentation in cancer remain largely obscure (Heitzer et al., 2020). Using digital droplet PCR, we show that cfDNA lengths are more variable across samples in open-chromatin regions compared to the pericentromeric closed chromatin locus in both colorectal cancer patients and healthy individuals. This complements the previously reported genome-averaged increase in the variance of cancer-derived cfDNA fragments (Cristiano et al., 2019). At the same time, we observed an increase in the relative number of shorter cfDNA fragments in CRC patients independent of chromatin state suggesting that cfDNA shortening in cancer is not mediated solely by cancer-related changes in chromatin accessibility to nucleases. Taken together, these results may indicate the presence of longer and more stable cfDNA fragments in the locus with a steady nucleosomal structure in contrast to OCRs and support the evidence of some cfDNA shortening in cancer patients regardless of chromatin state. However, the limited number of genomic loci analyzed with a sole ddPCR approach due to the limited cfDNA availability restricts generalization of these findings and further verification by alternative methods is crucial. Other limitations of the experimental design include an absence of tumor fraction estimations for CRC samples and a lack of samples from patients with diverse cancer types and benign lesions in the studied cohort. Moreover, there are some intrinsic limitations of PCR including inability to accurately quantify GC-rich matrices or tumor-derived fragments that are either too long or too short due to the fixed sizes of target amplicons. The fragility of cfDNA ends and technical variability of blood sample processing require careful evaluation of purified cfDNA samples prior to downstream analysis (Meddeb et al., 2019). We describe a convenient qPCR system for the detection of HMW DNA in cfDNA samples that requires low DNA input. Rapid screening with similar systems based on the differences in DNA size might become a standard step of cfDNA quality control workflow in the future. However, given the abovementioned complexity of cfDNA size profile, a fraction of HMW molecules in a sample not necessarily indicate an artificial contamination. It is also possible that longer molecules released from cells after blood draw may still to some extent be degraded by nucleases that retain activity in tube. This suggests more studies of cfDNA kinetics and release mechanisms. To conclude, our experimental evidence supports the prospects of ddPCR for the analysis of cfDNA fragment size in addition to the detection of somatic mutations and CNVs in cancer patients. We suggest considering a more broad involvement of quantitative PCR-based methods in cfDNA fragmentomics. If the acceptable performance is achieved the associated reduction of assay costs and run times will facilitate the transition to clinical use.

## Data Availability

The raw data supporting the conclusion of this article will be made available by the authors, without undue reservation.
