# Supplementary material for: Application of PCR-based approaches for evaluation of cell-free DNA fragmentation in colorectal cancer
Source: Front Mol Biosci. 2023 Mar 27;10:1101179. doi: 10.3389/fmolb.2023.1101179 (PMC10083340; doi:10.3389/fmolb.2023.1101179)
Supplement: Supplementary file 1 [file DataSheet2.PDF]

Supplementary qPCR data

The Supplementary **testexp.rdml** file contains a qPCR experiment with two targets called *Short* (106 bp – FAM) and *Long* (612 bp – HEX). The first part of the samples is a panel of standard samples with known mass fractions of HMW DNA: 50%, 25%, 5%, and 1%. The second part is the experimental samples with high and low rates of HMW DNA. To calculate the contamination score we set ΔΔCt calculation (Short target – reference, and 50% – control sample). Thus, we calculated “expression” – difference between control and unknown samples. For standard samples these were 50% for 50%, 24% for 25%, 8% for 5%, 2% for 1%. Such calculated results correspond to declared mixture. Difference between control and real samples also in consistency with theory (ratio 59%, 46 for highly contaminated, and 7%, 4% for lowly contaminated).

R code to analyze the **testexp.rdml** file:

```
library(tidyverse)
library(RDML)
library(ddCt)
rdml <- RDML$new("testexp.rdml")

cqTbl <- rdml$AsTable(cq = data$cq)

ddCtInput <- cqTbl %>%
  filter(!is.na(target.dyeId) & sample != "NTC") %>%
  select(Sample = sample, Detector = target, Ct = cq) %>%
  InputFrame()
ddCtResult <- ddCtExpression(ddCtInput,
                             calibrationSample = "50%",
                             housekeepingGenes = "Short") %>%
  ddCt()
ContaminationPercentage <-
  tibble(Sample = colnames(ddCtResult),
         ContPerc = ddCtResult[1, ]) %>%
  mutate(ContPerc = paste(round(2 ^ (-1 * ContPerc) * 50), "%"))
ContaminationPercentage
```
